# Editorial

**Published:** 1868-09-01

**Authors:** 


### *EDITORIAL.*

### *Please Notice.*

Correspondents and visitors will please notice that the heterogeneous jumble of names, known as the *Chicago Directory* for 1868–9, locates the Editor's office and residence at *Rush Medical College.* He has the honor of an official connection with that College, but his office is at 71 South Dearborn street, and his residence at 503 Michigan Avenue.

The oppositions of previous years made the several Directories tolerably reliable, but the *union* of the present year has proved any thing but strength, except to the olfactories. It will be difficult for visitors to find their friends or acquaintance, save by tracing them out in the Directories of previous years.

### *The Reform School.*

We have only space remaining in the present number to call attention to the fact that, according to the circulars of the "*one school only in the United States*," and its exposition in the columns of a contemporary, the pretended increase of requirements previous to graduation is a pretense and nothing more. The sliding scale adopted, as it is said, *temporarily*, can easily be seen to be a mere farce. The *status in quo* remains as before the Quixotic crusade of the "Phantom in Black."

### *The Nashville Imbroglio.*

In a late number we referred to the decision of Chancellor Shackleford with reference to Professors Eve, Jennings and Jones. It seems that the case afterwards went into chancery, and Judge Milligan issued a writ of *supersedeas*, etc., etc., the effect of which is to oust these gentlemen and sustain the new appointees. The writ issued, and Professors E., J. and J. are solemnly commanded to desist from all efforts to enforce Chancellor Shackleford's interlocutory order or decree, etc., etc. Whereupon Prof. Bowling issues a pronunciamento assuring the profession that everything is lovely, the new Faculty *en rapport* with right and justice, and harmony prevails. All of which we shall steadfastly believe until the next order from the courts. Meanwhile we can not conceive why the Nashville school is anxious to get rid of gentlemen so widely and favorably known as are Prof. Eve and his *confreres*. Do they drink too much? Or what is it? We await the issue of another circular to relieve our anxiety.

### *The Medical and Surgical History of the Rebellion,*

Being compiled in the Surgeon General's office, seems likely to be delayed in publication through the failure of sufficient appropriation therefor. Sixty thousand dollars were appropriated by the act of June 8th, 1868, for the publication of this history and the medical statistics of the Provost Marshal General's bureau—thirty thousand each. The superior cost of the first causing some trenching on the resources of the second, a special resolution was passed July 25th, ordering the balance of the appropriation to be devoted exclusively to the Provost Marshal's figures. This action is exceedingly to be regretted, for the profession were looking to the completion of the former work with very great expectations of benefit. It is to be hoped that some means will be afforded, during the coming session, to expedite this most important work.

### *Cook County Hospital, City of Chicago.*

WINTER TERM OF CLINICAL INSTRUCTION, 1868–9.—The regular course of Clinical Instruction in this Institution will commence on Friday, Oct. 2d, 1868, and continue for *nine months*.

The prosperity of this great public hospital, liberally supplied with the most adequate means for clinical and practical instruction, affords an extensive field for the study of every form of medical, surgical, and special disease.

During the year ending July 31st, 1868, over eleven hundred patients were treated in this hospital. Upwards of one hundred important surgical operations were performed.

In the lying-in department, there were ninety-eight births.

The large number of autopsies,—usually made in the presence of the class,—together with the pathological museum, give an excellent opportunity for the study of morbid anatomy.

The Dispensary, for out-patients, is organized so as to classify diseases, that every applicant shall receive the most thorough attention. This alone furnishes a large number of interesting cases.

Lectures will be delivered as follows: Surgical Clinics, with operations, and Medical Clinics, with examinations at the bedside, on Tuesdays and Fridays; Eye and Ear Clinics, with operations, and Clinic on Diseases of Women, on Saturdays, commencing invariably at half past one, and continuing two hours.

It is the purpose of the medical staff to use and develop the immense resources of the hospital for the promotion of medical education, and to add whatever is possible to the means and facilities for medical study attainable in Chicago. The object for which they will earnestly labor being an elevated standard of professional knowledge among the students, and such junior practitioners as may seek our city for this purpose.

Fee for admission to the Hospital, $5. Graduate practitioners free.

### *Lectures on the Eye and Ear.*

In addition to the course of lectures and operations on the Eye and Ear, embraced in the regular term of clinical instruction at the County Hospital, Dr. Hildreth will also deliver a *special course* on these organs.

The theory and uses of the ophthalmoscope, the analysis and treatment of the "accommodation," astigmatism, and other optical and mechanical defects of the eye will be dwelt upon.

The methods of exploring the ear will be fully demonstrated.

The number of interesting cases in the Eye and Ear Wards of the Hospital, ensures abundant opportunity for becoming familiar with ophthalmic and aural disease.

The Charitable Eye and Ear Clinic—a Free Dispensary, established by prominent citizens of Chicago, is likewise accessible to students.

The hospital ticket admits, without further charge, to all of the above.

### *O'Reilly Prize.*

Dr. John O'Reillv, of New York, having offered, through the New York Academy of Medicine, a Prize of Six Hundred Dollars for an Essay on the Physiology and Pathology of the Sympathetic or Ganglionic Nervous System, the Committee of Award appointed by the Council of the Academy, have adopted, with the concurrence of the Council, the following Regulations:

I.—The competing Essays shall be sent in to the Chairman of the Committe, Prof. J. C. Dalton, No. 101 East Twenty-third street, New York, on or before the First day of March, 1869.

II.—Each Essay shall be marked with some distinguishing device or motto, and accompanied by a sealed envelope bearing the same device or motto, and containing the name and address of the writer.

III.—The Essay selected by the Committee shall be transmitted by them, together with its accompanying envelope, to the Council of the New York Academy of Medicine, under whose direction the envelope shall be opened and the name of the writer announced at the first meeting of the Academy, in May, 1869.

IV.—This prize is open for universal competition.

V.—The Committee have a right to reject whatever does not come up to a proper standard of merit.

Alfred C. Post, M.D., *Pres't of the Academy, on behalf of the Board.*

*Committee of Awards.*—J. C. DALTON, M.D., Professor of Physiology in the College of Physicians and Surgeons, New York; A. FLINT, Jr., M.D., Professor of Physiology in the Bellevue Hospital Medical College, New York; ALFRED L. LOOMIS, M.D., Professor of the Institutes and Practice of Medicine in the University Medical College, New York.

☞ Medical journals are particularly requested to copy.

NEW YORK, *December*, 1867.

